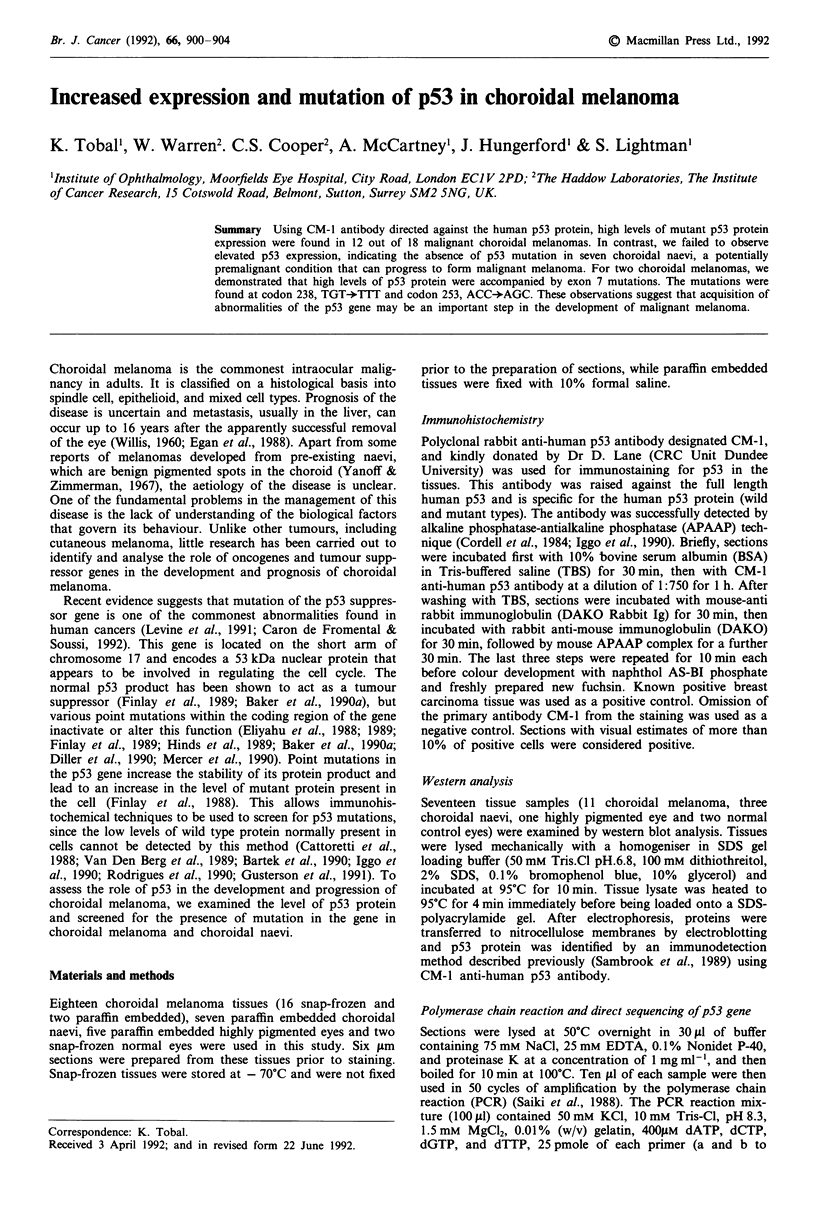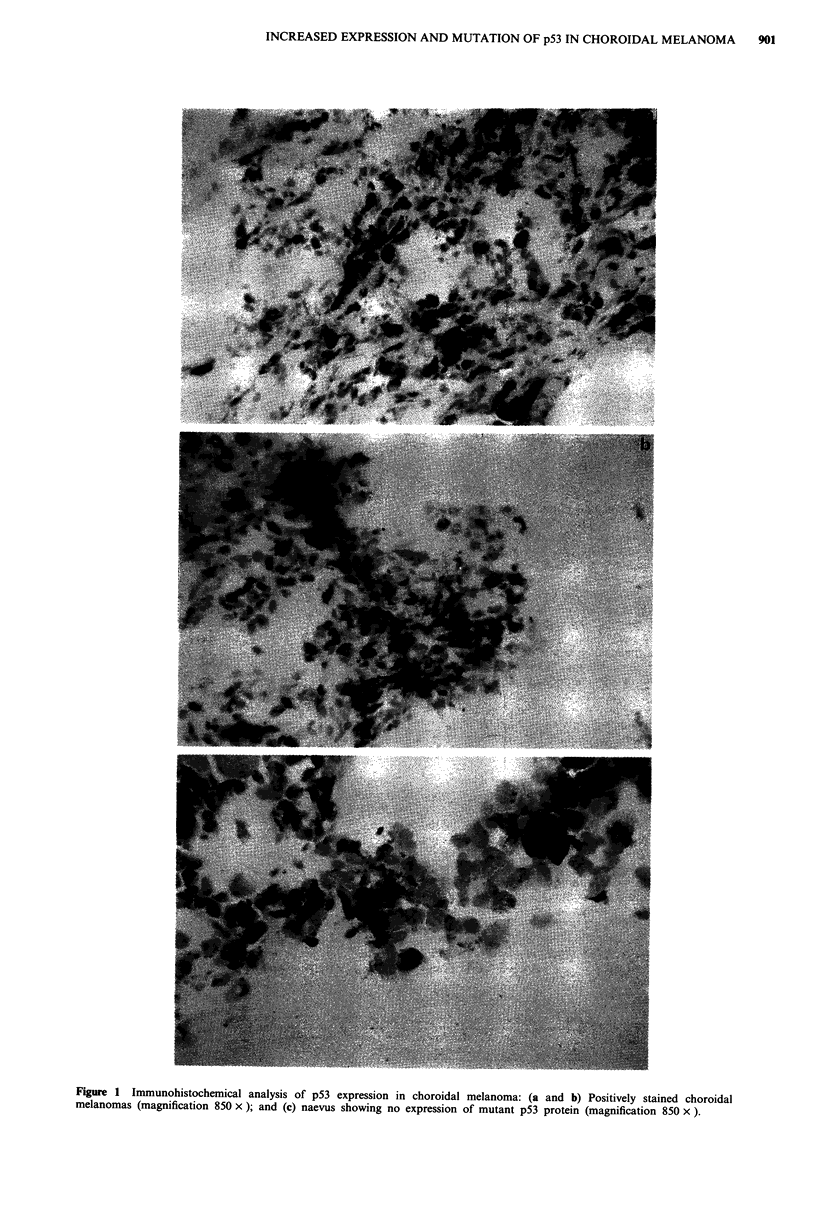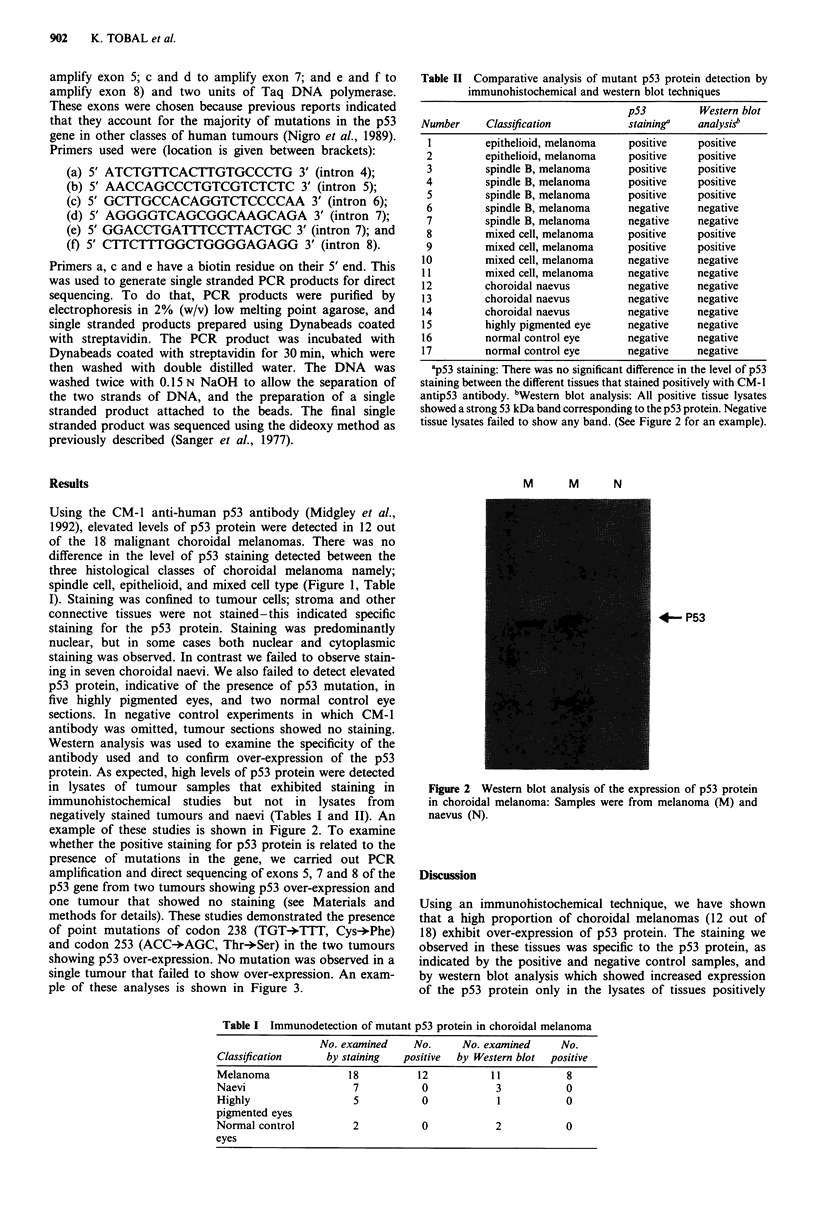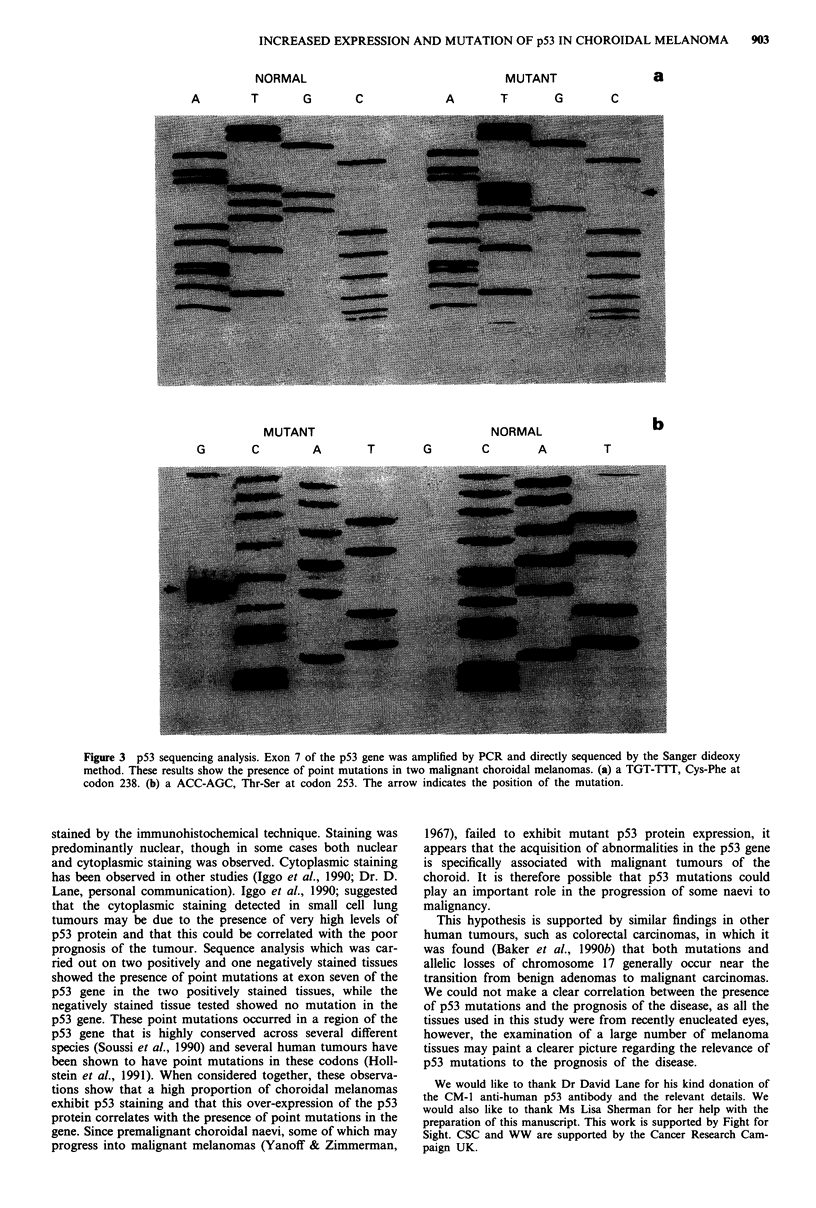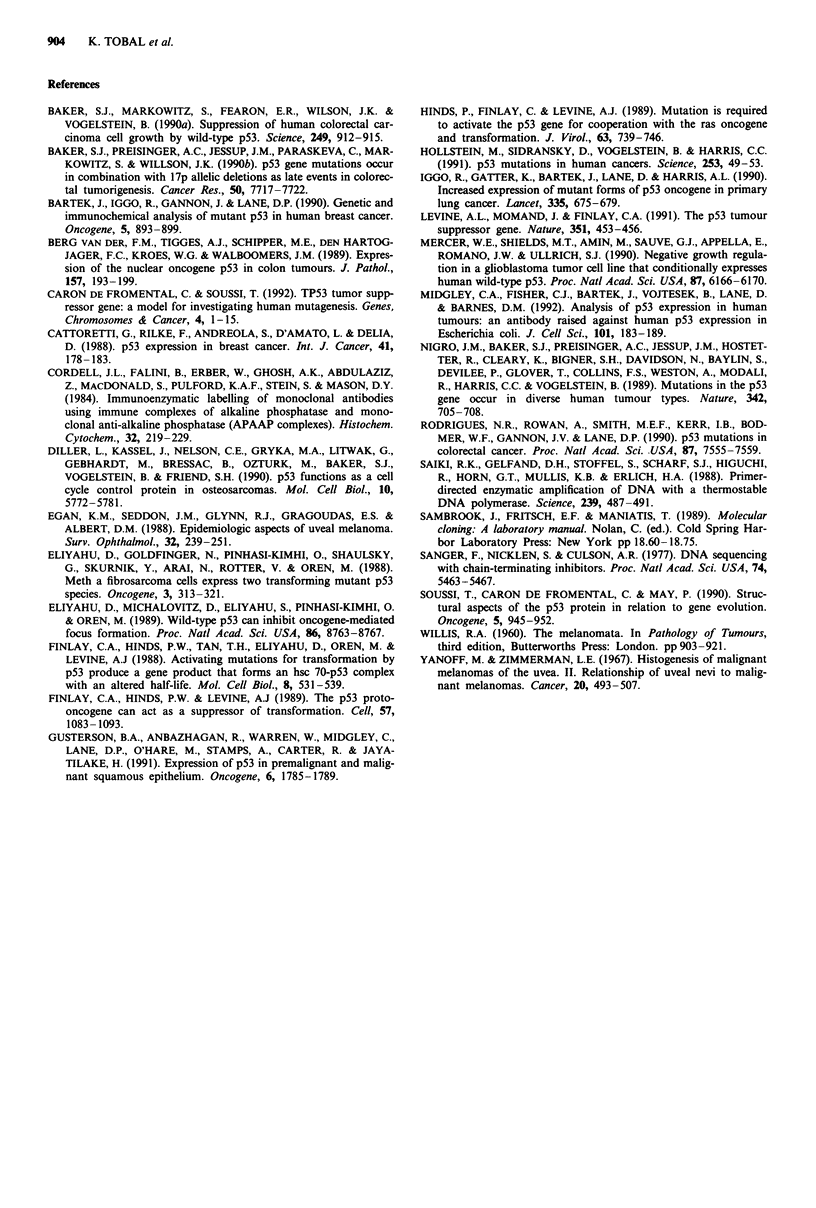# Increased expression and mutation of p53 in choroidal melanoma.

**DOI:** 10.1038/bjc.1992.382

**Published:** 1992-11

**Authors:** K. Tobal, W. Warren, C. S. Cooper, A. McCartney, J. Hungerford, S. Lightman

**Affiliations:** Institute of Ophthalmology, Moorfields Eye Hospital, London, UK.

## Abstract

**Images:**


Br. J. Cancer (1992), 66, 900–904 © Macmillan Press Ltd., 1992

### Increased expression and mutation of p53 in choroidal melanoma

K. Tobal[1], W. Warren[2]. C.S. Cooper[2], A. McCartney[1], J. Hungerford[1] & S. Lightman[1]

[1]*Institute of Ophthalmology, Moorfields Eye Hospital, City Road, London EC1V 2PD;* [2]*The Haddow Laboratories, The Institute of Cancer Research, 15 Cotswold Road, Belmont, Sutton, Surrey SM2 5NG, UK.*

**Summary** Using CM-1 antibody directed against the human p53 protein, high levels of mutant p53 protein expression were found in 12 out of 18 malignant choroidal melanomas. In contrast, we failed to observe elevated p53 expression, indicating the absence of p53 mutation in seven choroidal naevi, a potentially premalignant condition that can progress to form malignant melanoma. For two choroidal melanomas, we demonstrated that high levels of p53 protein were accompanied by exon 7 mutations. The mutations were found at codon 238, TGT→TTT and codon 253, ACC→AGC. These observations suggest that acquisition of abnormalities of the p53 gene may be an important step in the development of malignant melanoma.

Choroidal melanoma is the commonest intraocular malignancy in adults. It is classified on a histological basis into spindle cell, epithelioid, and mixed cell types. Prognosis of the disease is uncertain and metastasis, usually in the liver, can occur up to 16 years after the apparently successful removal of the eye (Willis, 1960; Egan *et al.*, 1988). Apart from some reports of melanomas developed from pre-existing naevi, which are benign pigmented spots in the choroid (Yanoff & Zimmerman, 1967), the aetiology of the disease is unclear. One of the fundamental problems in the management of this disease is the lack of understanding of the biological factors that govern its behaviour. Unlike other tumours, including cutaneous melanoma, little research has been carried out to identify and analyse the role of oncogenes and tumour suppressor genes in the development and prognosis of choroidal melanoma.

Recent evidence suggests that mutation of the p53 suppressor gene is one of the commonest abnormalities found in human cancers (Levine *et al.*, 1991; Caron de Fromental & Soussi, 1992). This gene is located on the short arm of chromosome 17 and encodes a 53 kDa nuclear protein that appears to be involved in regulating the cell cycle. The normal p53 product has been shown to act as a tumour suppressor (Finlay *et al.*, 1989; Baker *et al.*, 1990*a*), but various point mutations within the coding region of the gene inactivate or alter this function (Eliyahu *et al.*, 1988; 1989; Finlay *et al.*, 1989; Hinds *et al.*, 1989; Baker *et al.*, 1990*a*; Diller *et al.*, 1990; Mercer *et al.*, 1990). Point mutations in the p53 gene increase the stability of its protein product and lead to an increase in the level of mutant protein present in the cell (Finlay *et al.*, 1988). This allows immunohistochemical techniques to be used to screen for p53 mutations, since the low levels of wild type protein normally present in cells cannot be detected by this method (Cattoretti *et al.*, 1988; Van Den Berg *et al.*, 1989; Bartek *et al.*, 1990; Iggo *et al.*, 1990; Rodrigues *et al.*, 1990; Gusterson *et al.*, 1991). To assess the role of p53 in the development and progression of choroidal melanoma, we examined the level of p53 protein and screened for the presence of mutation in the gene in choroidal melanoma and choroidal naevi.

### Materials and methods

Eighteen choroidal melanoma tissues (16 snap-frozen and two paraffin embedded), seven paraffin embedded choroidal naevi, five paraffin embedded highly pigmented eyes and two snap-frozen normal eyes were used in this study. Six $\mu$m sections were prepared from these tissues prior to staining. Snap-frozen tissues were stored at $-70$°C and were not fixed prior to the preparation of sections, while paraffin embedded tissues were fixed with 10% formal saline.

### *Immunohistochemistry*

Polyclonal rabbit anti-human p53 antibody designated CM-1, and kindly donated by Dr D. Lane (CRC Unit Dundee University) was used for immunostaining for p53 in the tissues. This antibody was raised against the full length human p53 and is specific for the human p53 protein (wild and mutant types). The antibody was successfully detected by alkaline phosphatase-antialkaline phosphatase (APAAP) technique (Cordell *et al.*, 1984; Iggo *et al.*, 1990). Briefly, sections were incubated first with 10% bovine serum albumin (BSA) in Tris-buffered saline (TBS) for 30 min, then with CM-1 anti-human p53 antibody at a dilution of 1:750 for 1 h. After washing with TBS, sections were incubated with mouse-anti rabbit immunoglobulin (DAKO Rabbit Ig) for 30 min, then incubated with rabbit anti-mouse immunoglobulin (DAKO) for 30 min, followed by mouse APAAP complex for a further 30 min. The last three steps were repeated for 10 min each before colour development with naphthol AS-BI phosphate and freshly prepared new fuchsin. Known positive breast carcinoma tissue was used as a positive control. Omission of the primary antibody CM-1 from the staining was used as a negative control. Sections with visual estimates of more than 10% of positive cells were considered positive.

### *Western analysis*

Seventeen tissue samples (11 choroidal melanoma, three choroidal naevi, one highly pigmented eye and two normal control eyes) were examined by western blot analysis. Tissues were lysed mechanically with a homogeniser in SDS gel loading buffer (50 mM Tris.Cl pH.6.8, 100 mM dithiothreitol, 2% SDS, 0.1% bromophenol blue, 10% glycerol) and incubated at 95°C for 10 min. Tissue lysate was heated to 95°C for 4 min immediately before being loaded onto a SDS-polyacrylamide gel. After electrophoresis, proteins were transferred to nitrocellulose membranes by electroblotting and p53 protein was identified by an immunodetection method described previously (Sambrook *et al.*, 1989) using CM-1 anti-human p53 antibody.

### *Polymerase chain reaction and direct sequencing of p53 gene*

Sections were lysed at 50°C overnight in 30 $\mu$l of buffer containing 75 mM NaCl, 25 mM EDTA, 0.1% Nonidet P-40, and proteinase K at a concentration of 1 mg ml$^{-1}$, and then boiled for 10 min at 100°C. Ten $\mu$l of each sample were then used in 50 cycles of amplification by the polymerase chain reaction (PCR) (Saiki *et al.*, 1988). The PCR reaction mixture (100 $\mu$l) contained 50 mM KCl, 10 mM Tris-Cl, pH 8.3, 1.5 mM $MgCl_2$, 0.01% (w/v) gelatin, 400$\mu$M dATP, dCTP, dGTP, and dTTP, 25 pmole of each primer (a and b to

Correspondence: K. Tobal.
Received 3 April 1992; and in revised form 22 June 1992.

**Figure 1** Immunohistochemical analysis of p53 expression in choroidal melanoma: (**a** and **b**) Positively stained choroidal melanomas (magnification 850 ×); and (**c**) naevus showing no expression of mutant p53 protein (magnification 850 ×).

amplify exon 5; c and d to amplify exon 7; and e and f to amplify exon 8) and two units of Taq DNA polymerase. These exons were chosen because previous reports indicated that they account for the majority of mutations in the p53 gene in other classes of human tumours (Nigro *et al.*, 1989). Primers used were (location is given between brackets):

(a) 5′ ATCTGTTCACTTGTGCCCTG 3′ (intron 4);
(b) 5′ AACCAGCCCTGTCGTCTCTC 3′ (intron 5);
(c) 5′ GCTTGCCACAGGTCTCCCCAA 3′ (intron 6);
(d) 5′ AGGGGTCAGCGGCAAGCAGA 3′ (intron 7);
(e) 5′ GGACCTGATTTCCTTACTGC 3′ (intron 7); and
(f) 5′ CTTCTTTGGCTGGGGAGAGG 3′ (intron 8).

Primers a, c and e have a biotin residue on their 5′ end. This was used to generate single stranded PCR products for direct sequencing. To do that, PCR products were purified by electrophoresis in 2% (w/v) low melting point agarose, and single stranded products prepared using Dynabeads coated with streptavidin. The PCR product was incubated with Dynabeads coated with streptavidin for 30 min, which were then washed with double distilled water. The DNA was washed twice with 0.15 N NaOH to allow the separation of the two strands of DNA, and the preparation of a single stranded product attached to the beads. The final single stranded product was sequenced using the dideoxy method as previously described (Sanger *et al.*, 1977).

### Results

Using the CM-1 anti-human p53 antibody (Midgley *et al.*, 1992), elevated levels of p53 protein were detected in 12 out of the 18 malignant choroidal melanomas. There was no difference in the level of p53 staining detected between the three histological classes of choroidal melanoma namely; spindle cell, epithelioid, and mixed cell type (Figure 1, Table I). Staining was confined to tumour cells; stroma and other connective tissues were not stained–this indicated specific staining for the p53 protein. Staining was predominantly nuclear, but in some cases both nuclear and cytoplasmic staining was observed. In contrast we failed to observe staining in seven choroidal naevi. We also failed to detect elevated p53 protein, indicative of the presence of p53 mutation, in five highly pigmented eyes, and two normal control eye sections. In negative control experiments in which CM-1 antibody was omitted, tumour sections showed no staining. Western analysis was used to examine the specificity of the antibody used and to confirm over-expression of the p53 protein. As expected, high levels of p53 protein were detected in lysates of tumour samples that exhibited staining in immunohistochemical studies but not in lysates from negatively stained tumours and naevi (Tables I and II). An example of these studies is shown in Figure 2. To examine whether the positive staining for p53 protein is related to the presence of mutations in the gene, we carried out PCR amplification and direct sequencing of exons 5, 7 and 8 of the p53 gene from two tumours showing p53 over-expression and one tumour that showed no staining (see Materials and methods for details). These studies demonstrated the presence of point mutations of codon 238 (TGT→TTT, Cys→Phe) and codon 253 (ACC→AGC, Thr→Ser) in the two tumours showing p53 over-expression. No mutation was observed in a single tumour that failed to show over-expression. An example of these analyses is shown in Figure 3.

**Table II** Comparative analysis of mutant p53 protein detection by immunohistochemical and western blot techniques

| *Number* | *Classification* | *p53 staining*[a] | *Western blot analysis*[b] |
|---|---|---|---|
| 1 | epithelioid, melanoma | positive | positive |
| 2 | epithelioid, melanoma | positive | positive |
| 3 | spindle B, melanoma | positive | positive |
| 4 | spindle B, melanoma | positive | positive |
| 5 | spindle B, melanoma | positive | positive |
| 6 | spindle B, melanoma | negative | negative |
| 7 | spindle B, melanoma | negative | negative |
| 8 | mixed cell, melanoma | positive | positive |
| 9 | mixed cell, melanoma | positive | positive |
| 10 | mixed cell, melanoma | negative | negative |
| 11 | mixed cell, melanoma | negative | negative |
| 12 | choroidal naevus | negative | negative |
| 13 | choroidal naevus | negative | negative |
| 14 | choroidal naevus | negative | negative |
| 15 | highly pigmented eye | negative | negative |
| 16 | normal control eye | negative | negative |
| 17 | normal control eye | negative | negative |

[a]p53 staining: There was no significant difference in the level of p53 staining between the different tissues that stained positively with CM-1 antip53 antibody. [b]Western blot analysis: All positive tissue lysates showed a strong 53 kDa band corresponding to the p53 protein. Negative tissue lysates failed to show any band. (See Figure 2 for an example).

**Figure 2** Western blot analysis of the expression of p53 protein in choroidal melanoma: Samples were from melanoma (M) and naevus (N).

### Discussion

Using an immunohistochemical technique, we have shown that a high proportion of choroidal melanomas (12 out of 18) exhibit over-expression of p53 protein. The staining we observed in these tissues was specific to the p53 protein, as indicated by the positive and negative control samples, and by western blot analysis which showed increased expression of the p53 protein only in the lysates of tissues positively

**Table I** Immunodetection of mutant p53 protein in choroidal melanoma

| *Classification* | *No. examined by staining* | *No. positive* | *No. examined by Western blot* | *No. positive* |
|---|---|---|---|---|
| Melanoma | 18 | 12 | 11 | 8 |
| Naevi | 7 | 0 | 3 | 0 |
| Highly pigmented eyes | 5 | 0 | 1 | 0 |
| Normal control eyes | 2 | 0 | 2 | 0 |

**Figure 3** p53 sequencing analysis. Exon 7 of the p53 gene was amplified by PCR and directly sequenced by the Sanger dideoxy method. These results show the presence of point mutations in two malignant choroidal melanomas. (**a**) a TGT-TTT, Cys-Phe at codon 238. (**b**) a ACC-AGC, Thr-Ser at codon 253. The arrow indicates the position of the mutation.

stained by the immunohistochemical technique. Staining was predominantly nuclear, though in some cases both nuclear and cytoplasmic staining was observed. Cytoplasmic staining has been observed in other studies (Iggo *et al.*, 1990; Dr. D. Lane, personal communication). Iggo *et al.*, 1990; suggested that the cytoplasmic staining detected in small cell lung tumours may be due to the presence of very high levels of p53 protein and that this could be correlated with the poor prognosis of the tumour. Sequence analysis which was carried out on two positively and one negatively stained tissues showed the presence of point mutations at exon seven of the p53 gene in the two positively stained tissues, while the negatively stained tissue tested showed no mutation in the p53 gene. These point mutations occurred in a region of the p53 gene that is highly conserved across several different species (Soussi *et al.*, 1990) and several human tumours have been shown to have point mutations in these codons (Hollstein *et al.*, 1991). When considered together, these observations show that a high proportion of choroidal melanomas exhibit p53 staining and that this over-expression of the p53 protein correlates with the presence of point mutations in the gene. Since premalignant choroidal naevi, some of which may progress into malignant melanomas (Yanoff & Zimmerman, 1967), failed to exhibit mutant p53 protein expression, it appears that the acquisition of abnormalities in the p53 gene is specifically associated with malignant tumours of the choroid. It is therefore possible that p53 mutations could play an important role in the progression of some naevi to malignancy.

This hypothesis is supported by similar findings in other human tumours, such as colorectal carcinomas, in which it was found (Baker *et al.*, 1990*b*) that both mutations and allelic losses of chromosome 17 generally occur near the transition from benign adenomas to malignant carcinomas. We could not make a clear correlation between the presence of p53 mutations and the prognosis of the disease, as all the tissues used in this study were from recently enucleated eyes, however, the examination of a large number of melanoma tissues may paint a clearer picture regarding the relevance of p53 mutations to the prognosis of the disease.

We would like to thank Dr David Lane for his kind donation of the CM-1 anti-human p53 antibody and the relevant details. We would also like to thank Ms Lisa Sherman for her help with the preparation of this manuscript. This work is supported by Fight for Sight. CSC and WW are supported by the Cancer Research Campaign UK.

### References

BAKER, S.J., MARKOWITZ, S., FEARON, E.R., WILSON, J.K. & VOGELSTEIN, B. (1990*a*). Suppression of human colorectal carcinoma cell growth by wild-type p53. *Science*, **249**, 912–915.

BAKER, S.J., PREISINGER, A.C., JESSUP, J.M., PARASKEVA, C., MARKOWITZ, S. & WILLSON, J.K. (1990*b*). p53 gene mutations occur in combination with 17p allelic deletions as late events in colorectal tumorigenesis. *Cancer Res.*, **50**, 7717–7722.

BARTEK, J., IGGO, R., GANNON, J. & LANE, D.P. (1990). Genetic and immunochemical analysis of mutant p53 in human breast cancer. *Oncogene*, **5**, 893–899.

BERG VAN DER, F.M., TIGGES, A.J., SCHIPPER, M.E., DEN HARTOG-JAGER, F.C., KROES, W.G. & WALBOOMERS, J.M. (1989). Expression of the nuclear oncogene p53 in colon tumours. *J. Pathol.*, **157**, 193–199.

CARON DE FROMENTAL, C. & SOUSSI, T. (1992). TP53 tumor suppressor gene: a model for investigating human mutagenesis. *Genes, Chromosomes & Cancer*, **4**, 1–15.

CATTORETTI, G., RILKE, F., ANDREOLA, S., D'AMATO, L. & DELIA, D. (1988). p53 expression in breast cancer. *Int. J. Cancer*, **41**, 178–183.

CORDELL, J.L., FALINI, B., ERBER, W., GHOSH, A.K., ABDULAZIZ, Z., MACDONALD, S., PULFORD, K.A.F., STEIN, S. & MASON, D.Y. (1984). Immunoenzymatic labelling of monoclonal antibodies using immune complexes of alkaline phosphatase and monoclonal anti-alkaline phosphatase (APAAP complexes). *Histochem. Cytochem.*, **32**, 219–229.

DILLER, L., KASSEL, J., NELSON, C.E., GRYKA, M.A., LITWAK, G., GEBHARDT, M., BRESSAC, B., OZTURK, M., BAKER, S.J., VOGELSTEIN, B. & FRIEND, S.H. (1990). p53 functions as a cell cycle control protein in osteosarcomas. *Mol. Cell Biol.*, **10**, 5772–5781.

EGAN, K.M., SEDDON, J.M., GLYNN, R.J., GRAGOUDAS, E.S. & ALBERT, D.M. (1988). Epidemiologic aspects of uveal melanoma. *Surv. Ophthalmol.*, **32**, 239–251.

ELIYAHU, D., GOLDFINGER, N., PINHASI-KIMHI, O., SHAULSKY, G., SKURNIK, Y., ARAI, N., ROTTER, V. & OREN, M. (1988). Meth a fibrosarcoma cells express two transforming mutant p53 species. *Oncogene*, **3**, 313–321.

ELIYAHU, D., MICHALOVITZ, D., ELIYAHU, S., PINHASI-KIMHI, O. & OREN, M. (1989). Wild-type p53 can inhibit oncogene-mediated focus formation. *Proc. Natl Acad. Sci. USA*, **86**, 8763–8767.

FINLAY, C.A., HINDS, P.W., TAN, T.H., ELIYAHU, D., OREN, M. & LEVINE, A.J (1988). Activating mutations for transformation by p53 produce a gene product that forms an hsc 70-p53 complex with an altered half-life. *Mol. Cell Biol.*, **8**, 531–539.

FINLAY, C.A., HINDS, P.W. & LEVINE, A.J (1989). The p53 protooncogene can act as a suppressor of transformation. *Cell*, **57**, 1083–1093.

GUSTERSON, B.A., ANBAZHAGAN, R., WARREN, W., MIDGLEY, C., LANE, D.P., O'HARE, M., STAMPS, A., CARTER, R. & JAYATILAKE, H. (1991). Expression of p53 in premalignant and malignant squamous epithelium. *Oncogene*, **6**, 1785–1789.

HINDS, P., FINLAY, C. & LEVINE, A.J. (1989). Mutation is required to activate the p53 gene for cooperation with the ras oncogene and transformation. *J. Virol.*, **63**, 739–746.

HOLLSTEIN, M., SIDRANSKY, D., VOGELSTEIN, B. & HARRIS, C.C. (1991). p53 mutations in human cancers. *Science*, **253**, 49–53.

IGGO, R., GATTER, K., BARTEK, J., LANE, D. & HARRIS, A.L. (1990). Increased expression of mutant forms of p53 oncogene in primary lung cancer. *Lancet*, **335**, 675–679.

LEVINE, A.L., MOMAND, J. & FINLAY, C.A. (1991). The p53 tumour suppressor gene. *Nature*, **351**, 453–456.

MERCER, W.E., SHIELDS, M.T., AMIN, M., SAUVE, G.J., APPELLA, E., ROMANO, J.W. & ULLRICH, S.J. (1990). Negative growth regulation in a glioblastoma tumor cell line that conditionally expresses human wild-type p53. *Proc. Natl Acad. Sci. USA*, **87**, 6166–6170.

MIDGLEY, C.A., FISHER, C.J., BARTEK, J., VOJTESEK, B., LANE, D. & BARNES, D.M. (1992). Analysis of p53 expression in human tumours: an antibody raised against human p53 expression in Escherichia coli. *J. Cell Sci.*, **101**, 183–189.

NIGRO, J.M., BAKER, S.J., PREISINGER, A.C., JESSUP, J.M., HOSTETTER, R., CLEARY, K., BIGNER, S.H., DAVIDSON, N., BAYLIN, S., DEVILEE, P., GLOVER, T., COLLINS, F.S., WESTON, A., MODALI, R., HARRIS, C.C. & VOGELSTEIN, B. (1989). Mutations in the p53 gene occur in diverse human tumour types. *Nature*, **342**, 705–708.

RODRIGUES, N.R., ROWAN, A., SMITH, M.E.F., KERR, I.B., BODMER, W.F., GANNON, J.V. & LANE, D.P. (1990). p53 mutations in colorectal cancer. *Proc. Natl Acad. Sci. USA*, **87**, 7555–7559.

SAIKI, R.K., GELFAND, D.H., STOFFEL, S., SCHARF, S.J., HIGUCHI, R., HORN, G.T., MULLIS, K.B. & ERLICH, H.A. (1988). Primer-directed enzymatic amplification of DNA with a thermostable DNA polymerase. *Science*, **239**, 487–491.

SAMBROOK, J., FRITSCH, E.F. & MANIATIS, T. (1989). *Molecular cloning: A laboratory manual*. Nolan, C. (ed.). Cold Spring Harbor Laboratory Press: New York pp 18.60–18.75.

SANGER, F., NICKLEN, S. & CULSON, A.R. (1977). DNA sequencing with chain-terminating inhibitors. *Proc. Natl Acad. Sci. USA*, **74**, 5463–5467.

SOUSSI, T., CARON DE FROMENTAL, C. & MAY, P. (1990). Structural aspects of the p53 protein in relation to gene evolution. *Oncogene*, **5**, 945–952.

WILLIS, R.A. (1960). The melanomata. In *Pathology of Tumours*, third edition, Butterworths Press: London. pp 903–921.

YANOFF, M. & ZIMMERMAN, L.E. (1967). Histogenesis of malignant melanomas of the uvea. II. Relationship of uveal nevi to malignant melanomas. *Cancer*, **20**, 493–507.